# The evaluating self-management and educational support in severely obese patients awaiting multidisciplinary bariatric care (EVOLUTION) trial: rationale and design

**DOI:** 10.1186/1472-6963-13-321

**Published:** 2013-08-17

**Authors:** Raj S Padwal, Arya M Sharma, Miriam Fradette, Susan Jelinski, Scott Klarenbach, Alun Edwards, Sumit R Majumdar

**Affiliations:** 1 Department of Medicine, University of Alberta, Edmonton, Alberta, Canada; 2 Alberta Diabetes Institute, Edmonton, Alberta, Canada; 3 Applied Sciences and Research, Alberta Health Services, Calgary, Alberta, Canada; 4 Department of Medicine, University of Calgary, Calgary, Alberta, Canada; 5 2F1.26 Walter C. Mackenzie Health Sciences Centre, 8440-112th Street, Edmonton T6G 2B7, Alberta, Canada

**Keywords:** Bariatric care, Wait list, Severe obesity, Randomized controlled trial, Self management

## Abstract

**Background:**

In Canada, demand for multidisciplinary bariatric (obesity) care far outstrips capacity. Consequently, prolonged wait times exist and contribute to substantial health impairments.

A supportive, educational intervention (with in-person and web-based versions) designed to enhance the self-management skills of patients wait-listed for multidisciplinary bariatric medical and surgical care has been variably implemented across Alberta, Canada. However, its effectiveness has not been evaluated. Our objectives were: 1. To determine if this program improves clinical and humanistic outcomes and is cost-effective compared to a control intervention; and 2. To compare the effectiveness and cost-effectiveness of in-person group-based versus web-based care. We hypothesize that both the web-based and in-person programs will reduce body weight and improve outcomes compared to the control group. Furthermore, we hypothesize that the in-person version will be more effective but more costly than the web-based version.

**Methods/Design:**

This pragmatic, prospective controlled trial will enrol 660 wait-listed subjects (220 per study arm) from regional bariatric programs in Alberta and randomly assign them to: 1. an in-person, group-based intervention (9 modules delivered over 10 sessions); 2. a web-based intervention (13 modules); and 3. controls who will receive mailed literature. Subjects will have three months to review the content assigned to them (the intervention period) after which they will immediately enter the weight management clinic. Data will be collected at baseline and every 3 months for 9 months (study end), including: 1. Clinical [5% weight loss responders (primary outcome), absolute and % weight losses, changes in obesity-related comorbidities]; 2. Humanistic (health related quality of life, patient satisfaction, depression, and self-efficacy); and 3. Economic (incremental costs and utilities and cost per change in BMI assessed from the third party health care payor perspective) outcomes. Covariate-adjusted baseline-to-nine-month change-scores will be compared between groups for each outcome using linear regression for continuous outcomes and logistic regression for dichotomous ones.

**Discussion:**

Our findings will determine whether this intervention is effective and cost-effective compared to controls and if online or in-person care delivery is preferred. This information will be useful for clinicians, health-service providers and policy makers and should be generalizable to similar publically-funded bariatric care programs.

**Trial registration:**

Trial Identifier: NCT01860131

## Background

### Economic and public health burden of severe obesity

Obesity, currently affecting 24% of adult Canadians [1] is a chronic medical condition responsible for considerable morbidity [2], premature mortality [3], and impaired quality of life [4]. Obesity is most often defined according to body mass index (BMI). BMI levels of 30–34.9 kg/m^2^, 35–39.9 kg/m^2^ and over 40 kg/m^2^ correspond to Class I, II and III obesity, respectively with BMI levels of 18.5-24.9 kg/m^2^ considered normal. Obesity is considered the most costly of all common chronic medical conditions and, [5] in Canada, comprises up to 12% ($11 billion) of total health care expenditures [6]. For all of these reasons, obesity has become a central focus of and priority for clinicians, policymakers, health care administrators, regional health authorities and health care funders.

Severe obesity (defined herein as a BMI ≥ 35 kg/m^2^) currently affects 9% of Canadians. It is the fastest growing obesity subclass, having tripled in prevalence from 1978–79 to 2007–09 [1,7]. Severe obesity increases the risk of type 2 diabetes by more than 8-fold; [2] reduces life expectancy by 5–13 years; [8] increases health care expenditures by 50-200%; [9] and dramatically reduces quality of life [4].

### Treatment of severe obesity

#### Intensive lifestyle modification

Intensive lifestyle modification (diet, exercise ± behavioural modification) with a goal to reduce weight by 5-10% over 6–12 months is recommended as preferred initial therapy and should be delivered in a multidisciplinary setting [10]. Behavioural modification involves specific techniques to increase self-monitoring and self-efficacy. Examples include food journaling, goal setting, recognizing and addressing weight loss barriers (such as maladaptive eating behaviours), managing stress, increasing motivation and reframing negative emotions into positive ones [11]. Multidisciplinary teams are typically comprised of nurses, dietitians, physicians, mental health experts, physiotherapists and occupational therapists. One-year weight reductions in clinical trials examining intensive lifestyle modification average 6% of initial body weight (with most weight loss occurring within the first 3–6 months) and this degree of weight loss is associated with clinically significant improvements in cardiovascular risk factors (blood pressure, lipids, glycemic control) and reductions in diabetes incidence [10,12]. In Canada, publicly funded care for severely obese individuals is often delivered within multidisciplinary bariatric specialty clinics because many primary care settings lack the required support of the allied health team and infrastructure [13].

#### Antiobesity pharmacotherapy

Adding orlistat, a pancreatic lipase inhibitor and the only antiobesity drug approved in Canada, to lifestyle modification results in an additional ~3 kg of weight loss on average [14]. However, orlistat frequently leads to gastrointestinal adverse effects, has poor long-term persistence rates, and is rarely used [15].

#### Bariatric surgery

In Canada, bariatric surgery is currently indicated in patients with BMI levels of ≥ 40 kg/m^2^ or BMI levels of ≥ 35 kg/m^2^ with a major medical complication (e.g., hypertension, diabetes, sleep apnea) who are refractory to non-surgical therapy [16]. Bariatric procedures either involve stomach restriction alone or combined restriction plus intestinal diversion [17]. Surgery reduces weight by 33% after 2–3 years; [18] mortality rates by 29% after 15 years; [19] and leads to resolution or improvement of type 2 diabetes, hypertension, dyslipidemia and sleep apnea in 70-86% of cases [18]. However, surgery can lead to serious complications including rates of perioperative death of 0.1-0.5%, a 10% rate of serious complication and long-term nutrient deficiencies such as symptomatic B12 or iron deficiency [20]. Thus, the benefit-risk ratio must be carefully assessed and patients require multidisciplinary care to prepare for surgery and lifelong multidisciplinary follow-up and monitoring thereafter [21].

### Demand for multidisciplinary bariatric specialty care in Canada

Demand–supply gaps for multidisciplinary bariatric care exist in many jurisdictions in Canada (and elsewhere) and this has resulted in protracted wait times. As most multidisciplinary specialty bariatric programs in Canada deliver bariatric surgical care, the available published data mostly focus upon surgical wait times. Estimated average wait times for surgical procedures in programs across Canada are over 5 years [22]. Of the ~1.5 million Canadians considered eligible for bariatric surgery, only 0.1% receive the procedure annually [23]. Demand–supply gaps and wait times in other countries with publicly funded health care, such as the United Kingdom, are similarly prolonged [24-26].

### Bariatric specialty care in Alberta, Canada

The Edmonton Weight Wise program, established in 2005, was the first large scale, multidisciplinary bariatric program in Alberta. Weight Wise delivers integrated, patient-focused, evidence-based care to the Edmonton Zone of Alberta Health Services (AHS). This region is one of the largest integrated health delivery systems in Canada, serves a catchment population of 1.6 million residents, and has an annual healthcare budget of two billion dollars.

Weight Wise includes a central, region-wide, single-point-of-access referral system. The Adult Specialty Clinic offers intensive multidisciplinary medical/surgical bariatric care to patients with BMI levels of ≥ 35 kg/m^2^ (estimated to be at least 125 000 individuals within the region). Patients are referred for both medical and surgical management (i.e., not all patients are referred specifically for surgery). Approximately 850 new referrals are processed annually and 300 bariatric surgeries are performed.

Wait times for Weight Wise have fluctuated over time. In 2008, approximately 2000 patients were wait-listed for entry and the average wait time was at its peak at about 2 years. Currently (2013), patients wait 3–4 months before their initial assessment.

The Weight Wise model has been expanded and nascent multidisciplinary bariatric specialty programs have been established in other sites throughout Alberta (Calgary, Red Deer, Medicine Hat, Grande Prairie). These programs currently have wait times that are similar to or longer than waits for Edmonton Weight Wise, ranging from 2 to 6 months.

### Health consequences of protracted waits for bariatric specialty care

The health consequences of protracted waits for bariatric care have not been well studied. To our knowledge, only cross-sectional analyses have been published to-date. The findings suggest that self-reported health is markedly impaired in subjects wait-listed for bariatric care.

In 150 severely obese patients wait-listed for entry into Edmonton Weight Wise who had been waiting for 64±76 days (i.e., only recently added to the wait list), the mean health status, measured using a visual analogue score, (VAS) score was 49 out of 100. This is markedly lower than mean VAS scores for community-dwelling Albertans (85), patients with diabetes (66) and patients with chronic obstructive pulmonary disease (65) [27]. Two thirds (62%) of patients agreed or strongly agreed that waiting was contributing to physical, mental and financial deterioration and 86% also reported worsening of physical symptoms over time [27].

A Norwegian study in 128 severely obese patients reported that mental and physical Short Form-12 (SF-12) component scores were markedly lower than population norms [28]. Another study concluded that the HRQL of 75 wait-listed Finnish patients measured using the generic 15D instrument was ‘significantly worse-off’ compared to the general population [29].

In aggregate, these findings indicate a substantial and heretofore largely unrecognized and under-appreciated degree of health impairment in patients wait-listed for bariatric care. Importantly, patients report that protracted waits are directly contributing to deteriorations in quality of life and health. Confirmation of these findings using controlled, observational study designs is required.

### Supportive interventions to educate and increase the self-management skills of patients awaiting bariatric care

To our knowledge, no prior published randomized studies have examined interventions in patients wait-listed for bariatric care. Interventions to mitigate suffering, improve health, help patients to cope with extended waits, and ready patients for more intensive medical and surgical multidisciplinary care are needed but should also be rigorously evaluated to ensure that they are worthwhile. Notably, nearly 75% of wait-listed subjects in Edmonton Weight Wise are interested in receiving supportive care as they wait [27].

To support wait-listed patients as they wait for entry into the Weight Wise Adult Clinic, the Weight Wise Clinical Module (WWCM) program consisting of 9 modules (10 sessions) was initiated in 2008 (Table 1). These non-mandatory sessions are delivered in a group format by a multidisciplinary team at community health centers. All patients wait-listed for the Adult Clinic are eligible to attend free-of-charge and, typically, most sessions are attended over a 3–6 month time frame. The WWCM program is designed to educate patients regarding proper diet and exercise and improve weight management skills, with an emphasis on increasing self-management, enhancing self-efficacy and identifying/overcoming barriers to success. In addition to providing supportive care to wait-listed patients, WWCM is designed to prime patients for weight management success once they enter the Adult Clinic. Since the program was started in Edmonton, we estimate that over half of patients participated to some degree, although most did not attend all sessions. Thus, even though the program was free and available, in the majority of patients there has been variable uptake of WWCM.

**Table 1:** Weight wise community module: in-person program

**Topic**	**Content**
**Module one - getting started: planning for success**	Learn about obesity (what is it, what can cause it, what are some treatment choices).
	Learn ways to record what you eat, how active you are and how you are feeling emotionally.
	Learn about goal setting and how to deal with barriers.
**Module two – lifestyle change: a toolkit for success**	Learn how to make and keep healthy lifestyle changes.
	Learn strategies such as prioritizing, journaling, problem solving, thinking skills and goal setting.
**Module three - finding balance: the role of calories in weight management**	Learn about the top 5 ways to lower calories and practice strategies in class.
**Module four - managing hunger and appetite**	Explore the difference between hunger and appetite.
	Practice techniques to manage appetite triggers and social pressures.
**Module five - moving matters – including physical activity in your day**	Explore the benefits and barriers to being more active.
	Receive tips on how to get more physically active.
	Set your own personal activity goals.
**Module six - nutrition: the truth about what works in weight management**	Evaluate what you are eating and learn strategies that can help lower your calories.
	Explore how meal patterns, food choices and portion size affect calorie intake.
	Learn about which foods can help you manage your weight.
**Module seven - nutrition: i know i should eat healthy, but how?**	Leave with tips on how to put your nutrition knowledge into practice.
	Explore the 4 P’s: **P**lan, **P**urchase, **P**repare and **P**ack.
**Module eight- nutrition: eating away from home and during special occasions**	Learn how buffets, parties, vacation and holiday eating can affect calorie intake.
	Leave with strategies to minimize extra calories when eating away from home and during special occasions.
**Module nine - minding stress: effectively reduce and manage the stress in your life (two consecutive sessions)**	Learn about all the different reasons why people eat.
	Learn how to cope with emotional eating.
	Learn how to feel more in control of your eating.

The evidence-based curriculum [10] stresses healthy eating; gives practical tips to increase physical activity; teaches basic behavioural modification techniques such as goal setting and self-monitoring; and includes strategies for dealing with stress and maladaptive eating behaviours such as emotional eating. Each module is 2.5 hours in length and is led by the appropriate content expert(s) from a team of 4 registered nurses (3.2 FTE), 1 Canadian Society for Exercise Physiology (CSEP) specialist, 1 MSc psychologist and 1 registered dietician.

In 2012, the WWCM program was expanded to the other bariatric specialty care sites in Alberta. Notably, in the Calgary Region, a web-based version of the modules is also offered online, with similar content to that delivered in the in-person group sessions (Table 2). A comprehensive evaluation of the benefits and costs of the WWCM program has not been performed and is needed. In addition the optimal method of delivering WWCM content (i.e., group sessions versus online) is not known.

**Table 2:** Weight wise community module: web-based program

**Module**	**Content**
1a	Getting Started: Planning for Success
1b	Getting Started: Benefits and Challenges
1c	Getting Started: Skills for Weight Management Success
2a	Finding Balance: The Role of Calories in Weight Management – Calories and Diets
2b	Finding Balance: The Role of Calories in Weight Management – Top 5 Calorie Culprits & Tips for Reducing Calories
3a	Managing Hunger and Appetite: Managing Hunger
3b	Managing Hunger and Appetite: Controlling Your Appetite
4a	Moving Matters: How does moving matter?
4b	Moving Matters: Help me get moving! I’m ready!
5a	More on Nutrition: Transform Your Eating for Weight Loss
5b	More on Nutrition: Meal Planning Tips and Label Reading
5c	More on Nutrition: Eating Out and Special Occasions
6	A Good Night’s Sleep

If the program in any format is efficacious and cost-effective, we anticipate that other Canadian provinces and countries coping with protracted bariatric care wait times will consider implementing similar programs. Conversely if the WWCM program does not improve outcomes or is cost-prohibitive, the resources currently used for this program should be redeployed towards other bariatric care initiatives and alternate strategies for supporting wait-listed patients will need to be considered.

## Objectives

The EVOLUTION Trial is designed to:

1. Assess the clinical effectiveness of the WWCM program in reducing weight, improving obesity-related comorbidity and decreasing Adult Clinic attrition rates compared to controls.

2. Determine if the WWCM program improves humanistic outcomes including health-related quality of life, patient satisfaction, self-efficacy and readiness-to-change compared to controls.

3. Determine the incremental cost and cost-effectiveness of the WWCM program.

4. Compare the in-person delivery of WWCM content to online delivery in terms of efficacy and cost-effectiveness.

In aggregate, these objectives will assess the program’s impact on a comprehensive range of outcomes important to patients, providers, and policy-makers. Because the WWCM program is intended to prime patients for success as they receive care in the Adult Clinic, the primary outcomes will be measured prior to attending WWCM modules and then repeated six months after entry into the Adult Clinic.

We hypothesize that both online and in-person delivery of the WWCM intervention will reduce body weight and improve clinical and humanistic outcomes at 9 months compared to the control group. Furthermore, we hypothesize that the in-person version of the intervention will be more effective (as a consequence of greater interpersonal contact) but also more costly than the on-line version. Although we hypothesize that the program will be beneficial, this is by no means certain. It is possible that outcomes in the groups receiving the WWCM intervention will be no better than controls, only marginally better, or improved but not in a cost-effective manner. This underscores the need for rigorous program evaluation to ensure that current program delivery - as well as potential future expansion - is warranted.

## Methods/Design

### Study design

This 9-month pragmatic, prospective, randomized controlled trial will enrol consecutive, consenting severely obese patients newly wait listed for the adult bariatric specialty care. Subjects will be randomly assigned one of three groups:

1. Weight Wise Community Modules delivered in-person: 9 modules delivered over 10 group sessions.

2. Weight Wise Community Modules delivered on the web: 13 modules with similar content to the in-person sessions

3. Mailed Educational Pamphlets including Canada’s Guide to Healthy Living (controls).

Each study arm will be comprised of 220 patients (660 total).

### Subjects

Inclusion criteria (all criteria must be met):

1. BMI ≥ 35 kg/m^2^

2. Newly wait-listed for entry into a adult (age > 18 years) bariatric specialty clinic

Exclusion criteria (any one sufficient to exclude):

1. Completed more than 4 Weight Wise Community Modules (web-based or group session) in previous 3 months

2. Pregnant female

3. Unable to read/write/comprehend English

4. Unable to access the web

5. Unable or unwilling to attend in-person module sessions

6. Uncontrolled severe personality disorder, active psychosis, active substance dependence and/or major cognitive impairment

7. Deemed unsuitable by the study investigators

8. Participation in concurrent trial related to obesity management

9. Resides greater than 1 hour driving time to Weight Wise clinic

10. Declined to participate

### Procedures

Two clinics in Alberta, located in Edmonton and Red Deer, will participate in the study, although we have the potential to expand to other bariatric speciality care sites in Calgary, Grande Prairie and Medicine Hat (up to 5 sites total) if needed. Newly wait-listed patients will be contacted by telephone and invited to participate. They will be assessed in-person within 2 weeks of telephone contact to confirm eligibility and provide written consent prior to randomization. Baseline data collection and group specific patient instructions will be provided at this visit. All study participants will be instructed to not begin any other new lifestyle modification or weight-loss related interventions during the first 3 months of the study.

Subjects will be contacted by telephone within 1 week to ensure those allocated to the WWCM have registered for the WWCM sessions (either in-person or web-based) and to address any questions or concerns. Those randomized to the WWCM program will have 3 months to complete the modules. Thereafter, all subjects, including controls, will immediately enter the weight management clinic in their zone to receive multidisciplinary bariatric care. The control group will only receive mailed literature that includes national dietary guidelines. They will receive a phone call at one week reminding them to review the written materials. Although strict patient-level blinding per se will not be possible, subjects will be given general information that the purpose of the study is to examine three different weight management delivery methods to wait-listed patients and more specific information on their assigned treatment group only.

### Randomization and allocation

Patients will be randomized 1:1:1 to WWCM in-person sessions or WWCM web-based sessions or mailed literature. Computer-generated randomization will be performed centrally and independently by the EPICORE center (http://www.epicore.ualberta.ca) to ensure allocation concealment from all research personnel; randomization will be stratified by participating study centre. Although clinic staff can not be blinded to allocation status, all outcome assessments will be performed by research assistants working independently from regular clinic staff.

### Data collection and outcome measures

**Figure 1:**
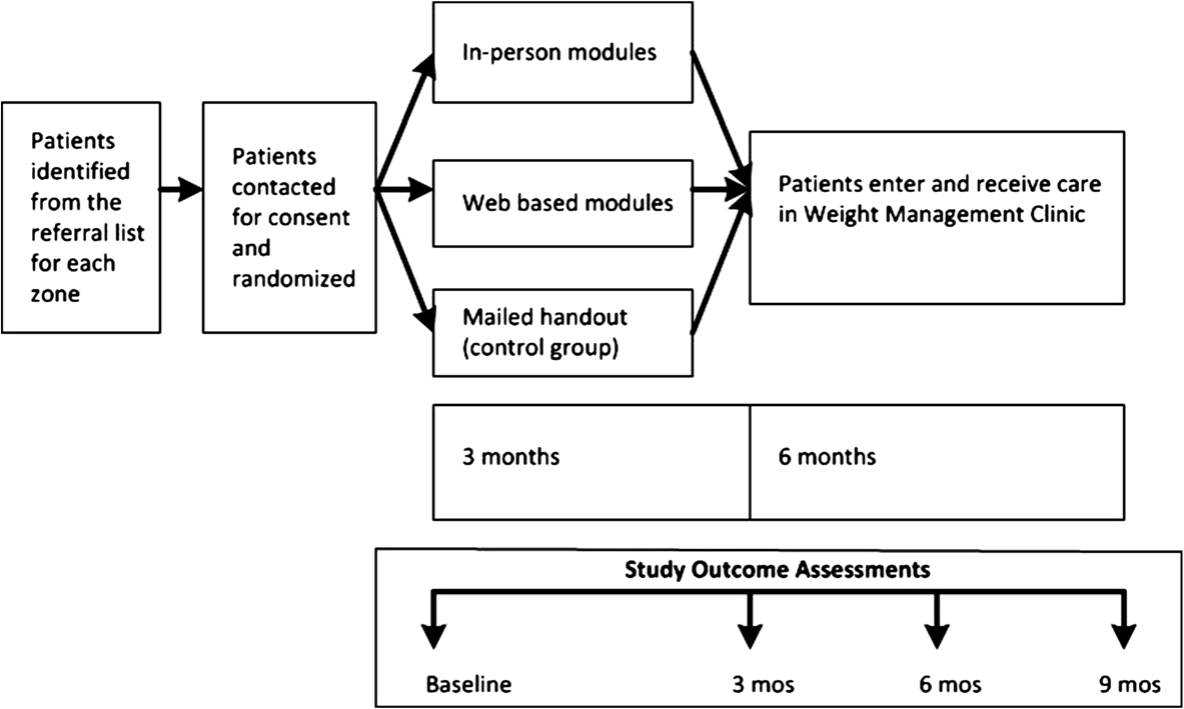
Study overview.

Unless otherwise indicated, data will be collected at baseline, and at 3, 6 and 9-months post-randomization (Additional file 1); the 9-month time point is the one of primary interest (see Figure 1). Study personnel will collect data using standardized case report forms (paper copies will be retained and securely stored) and data transferred on a daily basis to an electronic database using a secure, internet-based portal.

1. **Sociodemographic data (baseline only):** This includes age, sex, race, marital status, household income and employment status.

2. **Obesity-related comorbidities and past medical history:** includes blood pressure taken with a Watch BP® automated monitor (three readings simultaneously in each arm; first reading discarded and the latter two averaged; higher arm average used as the primary endpoint), fasting lipids and glucose, A1c, creatinine, alanine aminotransferase (ALT) smoking status and cardiovascular medications.

3. **Specific obesity-related variables:** weight, height (using a clinic based stadiometer), BMI.

4. **Quality of life and utility measurement:** Validated generic health related quality of life (SF-12 [35] and EQ-5D [30]) instruments will be used. Preference-based utilities will be measured using the EQ-5D.

5. **Satisfaction with medical care:** assessed using two questions from the Patient Satisfaction Questionnaire (PSQ) [31] that will be scored on a 5-point Likert Scale.

6. **Self-efficacy:** Obesity-specific self-efficacy will be measured using the validated Weight Efficacy Life-Style Questionnaire (WEL), a 20-item instrument that assesses self-efficacy across 5 situational factors (negative emotions, availability, social pressure, physical discomfort and positive activities) [32].

7. **Screening for Depression:** the Patient Health Questionnaire (PHQ-8), a validated screening tool for depression, will be used [33].

8. **Readiness-to-Change:** assessed using a a Visual Analogue scale ranging from 0 to 10.

9. **Attrition Rates (at 6 and 9 months only):** attrition rates within the multidisciplinary clinic will be assessed and compared between study arms.

10. **Costing Data:** Costing will adhere to the three-step micro-costing technique of identification, measurement, and valuation of resources. We will determine the overall and per-patient total and incremental costs of the two versions of the WWCM program and the control arm [34,35]. Start-up costs and on-going program delivery costs for each study arm will be tabulated. The cost per patient will be estimated using the current enrolment in the WWCM, as well as best- and worst-case scenarios (minimal and maximum session capacity) [34,35].

### Statistical analyses and sample size considerations

The primary outcome comparisons of interest are between the in-person WWCM intervention and controls and the online WWCM intervention and controls. Subsequent analyses will compare outcomes between the in-person and online interventions. No adjustment for multiple testing will be performed [36].

### Clinical endpoints

#### Outcomes

Assessment of clinical effectiveness will include examination of changes in dichotomous outcomes (proportion of patients achieving 5% and 10% weight loss, prevalence of diabetes, hypertension, dyslipidemia, and sleep apnea as defined by lab parameters, patient report and/or use of active treatment) and continuous outcomes (anthropometric indices, blood pressure, lipid profile, glycemic control).

#### Data analysis

First, variables will be examined descriptively and graphically, including assessments of temporal trends and tests of normality. Second, the 3-month and 9-month mean change from baseline measures in each outcome will be calculated and compared between cases and controls using unpaired t-tests for continuous outcomes and chi-squared tests for dichotomous ones. Third, multivariable predictors of the 9-month change in a given outcome will be identified using appropriately constructed and calibrated linear regression models for continuous outcomes or logistic regression models for dichotomous ones. Herein, we present a detailed example of our modelling strategies for body weight. First, the proportion of patients achieving 5% weight loss (5% responders, a value widely considered as a clinically worthwhile weight change) [10] and the mean 9-month change in weight will be calculated in each study arm. Second, chi-square tests will examine for significant differences between cases and controls in 5% response rates and unpaired t-tests will examine for significant differences in mean body weight change scores. Third, a multivariable logistic regression model for 5% responders and a linear regression model for mean body weight will be constructed to examine the independent effect of the WWCM program on the 9-month change in 5% response rate or mean body weight, adjusting for any statistically significant (P<0.1) or clinically important between group differences. Missing data for weight change will be addressed using a conservative ‘baseline-value carried forward’ approach and examined in a sensitivity analysis using group mean-imputation methods to confirm the robustness of results [37]. All analyses will be conducted according to the “intention to treat principle”. No interim analyses, for either efficacy or futility purposes, have been planned.

#### Sample size considerations

Our sample size estimation is based upon the desire to detect a 15% difference between the WWCM interventions and controls in the proportion of 5% weight responders with an alpha level of 0.05 and a power of 0.90. We assume that the enhanced control arm will result in a 5% weight loss in 20% of subjects (i.e., the control event rate = 20% at one year) [38]. Given these parameters, the minimal required sample size will be ~180 patients per arm or 540 patients total (http://statpages.org/proppowr.html). As losses to follow-up have reached 30% in previous obesity related trials; [39] we will also adjust for a 30% potential drop out rate. Therefore, our final estimated sample size will be 220 patients per arm, for a total sample size of 660 patients.

We chose a 5% weight loss threshold as the primary outcome measure of individual patient success (rather than using mean absolute body weight or a similar continuous parameter) based upon the recommendations of current consensus guidelines, which endorse this degree of weight loss as the minimum target to achieve clinically meaningful health benefits after 6–12 months of intervention [10]. We chose a 15% absolute difference between study arms as the minimum clinically worthwhile (important) difference (MCID) by consensus of clinical experts and trials methodologists and have used this type of consensus-based threshold definition in prior studies [40]. We felt that if the WWCM program produced less than a 15% improvement in weight loss [5% response] compared with controls it would not be worth continued funding - unless very compelling benefits on humanistic outcomes or cost-effectiveness were also demonstrated. We partly based our rationale for choosing this threshold on the opinions of prior consensus-based expert panels, that have proposed a 10% MCID for 1-year weight change patients with type 2 diabetes (while recognizing that weight reductions in type 2 diabetics are generally lower than non-diabetics) and a 15% MCID for statin uptake in patients with coronary disease [41,42].

### Humanistic endpoints

#### Outcomes

The instruments to be used to assess health related quality of life (SF-12, EQ-5D and PHQ-8), self-efficacy (WEL), depression (PHQ-8) and patient satisfaction (PSQ) are described above. The mental and physical domains of each instrument (where applicable) will be examined in subgroup analyses and (where available) compared with population-based normative data available for the Alberta population.

#### Data analysis

Descriptive analyses will be performed and data will be examined graphically and serial changes described. Because these are self-reported data collected every 3 months, we will use a “last-value carried forward” approach to handle missing data as our primary analytic strategy. For example, temporal trends in the SF-12 will be assessed graphically and using analysis of variance (ANOVA) as a test of trends over time. Multivariable, adjusted 9-month changes in continuous measures will be assessed using linear regression in a manner similar to that detailed for the clinical outcome analysis. Patient satisfaction will be categorized as a dichotomous variable [31] and multivariable predictors of deterioration in satisfaction will be determined in serially constructed models.

#### Sample size considerations

The sample size of 660 subjects has more than adequate power. For example, for a nine month change in SF-36 domains such as physical function, with baseline score of 60, SD 19 [43], a deterioration of 10 points, two-sided alpha=0.05, beta=0.90, and a 10% attrition rate, only 85 total patients would be required. Similarly, for an outcome such as satisfaction, with baseline measures of 70% satisfied with care, a 30% deterioration in satisfaction over 1 year, and otherwise similar power-related assumptions, 136 total patients are required.

### Economic endpoints

#### Outcomes

The total and incremental cost of WWCM (web-based and in-person) vs. controls (by program and per patient) will be determined and the incremental cost per QALY gained (cost-utility), and cost per incremental difference in BMI (cost-effectiveness) will be calculated.

#### Methods and analyses

A validated economic model of interventions in bariatric patients will be adapted to compare each version of the WWCM program vs. controls while adhering to recommended best practices for conduct of economic evaluation [18,44]. The model will be informed by primary data, including costing data, utility scores (EQ-5D) and changes in BMI in the 3 groups at 9-months. A 9-month time horizon and a public health care payer perspective will be used. No discounting of costs and benefits will be performed in the reference case (given the short time frame). Uncertainty and variability will be explored through sensitivity analysis, including one-way and probabilistic sensitivity analysis to determine if results are modified over a plausible range of model inputs (for example, 95% confidence intervals of differences in EQ-5D, or variability in cost per patient of the WWCM program). If differences in BMI are found between the treatment groups at 9-months, we will perform an exploratory lifetime analysis linking BMI at 9-months to longer term health outcomes (including probability of developing complications such as diabetes, sleep apnea, long-term quality of life, and risk of death) as conducted in our previous work [18,19,44,45].

### Preplanned subgroup analyses

Subgroup analyses will be conducted to explore differences in weight loss and humanistic outcomes according to baseline BMI, presence or absence of diabetes, age, sex, and diagnosis of depression or other mental illness.

### Ethical and other considerations

All subjects will provide written informed consent. The EVOLUTION trial protocol has been approved University of Alberta Research Ethics Board (PRO00031699) and it has received peer reviewed funding from the Canadian Institutes of Health Research (grant #267297). The trial has been formally registered at clinicaltrials.gov (NCT01860131).

## Discussion

In summary, EVOLUTION is a pragmatic randomized controlled trial that will determine if an existing intervention, the WWCM program, improves health outcomes in a cost-effective manner. In addition, if effective, the optimal care delivery method (in-person versus online) will be identified.

The population-based nature of the bariatric specialty clinics from which EVOLUTION subjects will be enrolled enhances generalizability because the structure of these clinics is similar to those found in other provinces within Canada. If EVOLUTION demonstrates that the WWCM program is effective and cost-effective, we anticipate that other programs will seek to implement similar interventions. If WWCM program has limited effectiveness or cost-effectiveness, it’s use should be re-evaluated and funding reallocated to other bariatric care initiatives. Thus, no matter the study findings, we feel that they will assist health-service providers and policy makers to better allocate use of finite resources.

EVOLUTION commenced enrolment in March 2013. Recruitment of all 660 subjects is expected by end-2015. Final study results are anticipated in 2016.

## Competing interests

EVOLUTION is funded by Canadian Institute for Health Research (CIHR) grant number 267297. The authors’ declare no competing interests with respect to this study.

RP, SWK, SRM and AMS are supported by an alternative funding plan from the Government of Alberta and the University of Alberta. AMS is supported by an Alberta Health Services Chair in Obesity Research and Management. SWK and SRM hold salary support awards from Alberta Heritage Foundation for Medical Research and Alberta Innovates-Health Solutions. SRM holds the Endowed Chair in Patient Health Management of the Faculties of Medicine and Dentistry and Pharmacy and Pharmaceutical Sciences, University of Alberta.

## Authors’ contributions

RP drafted the initial study concept and all authors contributed to the study design. RP wrote the initial draft of the protocol and all authors provided input into revisions and approved the final draft.

## Pre-publication history

The pre-publication history for this paper can be accessed here:

http://www.biomedcentral.com/1472-6963/13/321/prepub

## Supplementary Material

Additional file 1 Case Report Forms. Click here for file
