# Supplementary material for: The evaluating self-management and educational support in severely obese patients awaiting multidisciplinary bariatric care (EVOLUTION) trial: rationale and design
Source: BMC Health Serv Res. 2013 Aug 17;13:321. doi: 10.1186/1472-6963-13-321 (PMC3751526; doi:10.1186/1472-6963-13-321)
Supplement: Additional file 1 — Case Report Forms. [file 1472-6963-13-321-S1.pdf]

EVOLUTION

Evaluating Self-Management and Educational Support in Extremely Obese Patients Awaiting Multidisciplinary Bariatric Care

Screening ID [ ] - S [ ] [ ] [ ] [ ]
Site # Participant #

Participant Initials [ ] [ ] [ ]
First Middle Last

Date Screened _____ / _____ / __________
dd mm yyyy

Inclusion Criteria *(all criteria must be met)*

• BMI ≥35 kg/m^2	☐ Yes	☐ No
• Newly wait-listed for a provincial *Weight Wise Adult Clinic*	☐ Yes	☐ No
• Adult age ≥18 years	☐ Yes	☐ No

Exclusion Criteria *(any one is sufficient to exclude)*

• Completed more than 4 *Weight Wise* Community Modules *(web-based or group session)* in previous 3 months	☐ Yes	☐ No
• Pregnant female	☐ Yes	☐ No
• Unable to read/write/comprehend English	☐ Yes	☐ No
• Unable to access the web	☐ Yes	☐ No
• Unable or unwilling to attend in-person module sessions	☐ Yes	☐ No
• Untreated severe personality disorder, active psychosis, active substance dependence and/or major cognitive impairment	☐ Yes	☐ No
• Unsuitable study candidate *(as deemed by study team)*	☐ Yes	☐ No
• Participation in concurrent trial related to obesity management	☐ Yes	☐ No
• Resides >1 hour driving time of *Weight Wise Clinic*	☐ Yes	☐ No
• Declined to participate	☐ Yes	☐ No
If Yes, Age _____ yrs Sex: ☐ Male ☐ Female Weight ______ *(kg)* Height ______ *(cm)*		
• Unable to contact *(3 phone calls, no response within 48 hours)*	☐ Yes	☐ No
• Not contacted by Study Team	☐ Yes	☐ No

Eligibility

• Based on criteria above, is participant eligible for this study ☐ Yes ☐ No

If Yes, Schedule Baseline Visit → Proceed to ***Eligibility*** form

If No, Participant must be excluded

Form completed by ______________________ Signature ______________________ Date ____/____/_______
(please print name) dd mm yyyy

©2013 EPICORE Centre

Fax completed forms to EPICORE Centre (780) 492-6059 or 1-888-215-5474

EVOLUTION

Evaluating Self-Management and Educational Support in Extremely Obese Patients Awaiting Multidisciplinary Bariatric Care

Study ID [ ] - [ ][ ][ ]
Site # Randomization #

Participant Initials [ ][ ][ ]
First Middle Last

BMI

- Measure **actual** weight and height

**Weight**	________ kg	**Height**	________ cm	Reminder: Convert to meters to calculate BMI

**BMI** *(not for data entry)*	________ Weight in kg / ________ Height in m^2 = ________ BMI (kg/m^2)

Eligibility

- Based on criteria above, is participant **still eligible** for this study *(i.e. BMI ≥35 kg/m^2)* ☐ Yes ☐ No

If No, Participant must be excluded

→ Contact the EVOLUTION Study Coordinator

→ Fax this form to *EPICORE Centre at (780) 492-6059 or 1-888-215-5474*

Consent

- Was informed written consent received ☐ Yes ☐ No

If Yes, Proceed to ***Randomization*** form

If No, Participant must be excluded

→ Contact the EVOLUTION Study Coordinator

→ Fax this form to *EPICORE Centre at (780) 492-6059 or 1-888-215-5474*

Form completed by ____________________ (please print name) Signature ____________________ Date ____/____/________ dd mm yyyy

©2013 EPICORE Centre

Fax completed forms to EPICORE Centre (780) 492-6059 or 1-888-215-5474

EVOLUTION

Evaluating Self-Management and Educational Support in Extremely Obese Patients Awaiting Multidisciplinary Bariatric Care

Study ID [ ] - [ ][ ][ ]
Site # Randomization #

Participant Initials [ ][ ][ ]
First Middle Last

Randomization

Randomization Instructions:

Go to *EPICORE Centre* website: **https://**www.epicore.ualberta.ca
Click on 'Randomization Service'
Follow website instructions
Record participant's 'Randomization #' below

Date of Randomization ______ / ______ / ___________
dd mm yyyy

Randomization # [ ][ ][ ]

Treatment Assignment

- ☐ **In person-Module:** *Weight Wise* Community Modules delivered in person
- ☐ **Web-based Module:** *Weight Wise* Community Modules delivered via web
- ☐ **Control**: Educational pamphlets *(Canada's Guide to Healthy Living)*

- Complete ***Contact Information*** form

Form completed by ________________________ Signature ________________________ Date ____/____/________
(please print name) dd mm yyyy

©2013 EPICORE Centre

EVOLUTION

Evaluating Self-Management and Educational Support in Extremely Obese Patients Awaiting Multidisciplinary Bariatric Care

Study ID [ ] **-** [ ][ ][ ] **Participant Initials** [ ][ ][ ]

Site # Randomization # First Middle Last

Participant ______________________________, ____________________________________

Last Name First Name and Initial

____

Street Address Town/City Province

Postal Code, Email Address and PHN# to be entered into database

Postal Code ____________ **Email Address** ____

PHN#: __________________________

Telephone Number(s) Home ( ) _______ - _____________
Area code

Work ( ) _______ - _____________
Area code

Cell ( ) _______ - _____________
Area code

Preferred Contact Time A.M. ________ P.M. ________

Secondary Contact Person
(Close family member or friend – NOT living with the participant) __________________________ ___________________________

Last Name First Name

Relationship to Participant ____

Telephone Number: Home ( ) ______ - _________ Work ( ) ______ - _________
Area code Area code

Primary Physician Name ____

Telephone Number ( ) _______ - ____________ **Fax** ( ) _______ - ____________

Pharmacy Name ____

Telephone Number ( ) _______ - ____________ **Fax** ( ) _______ - ____________

©2013 EPICORE Centre

EVOLUTION

Evaluating Self-Management and Educational Support in Extremely Obese Patients Awaiting Multidisciplinary Bariatric Care

Study ID ☐ - ☐☐☐ (Site # / Randomization #) **Participant Initials** ☐☐☐ (First / Middle / Last)

Demographics

- Date of Baseline Visit _____ / _____ / __________ (dd / mm / yyyy)
- Date of Birth _____ / _____ / __________ (dd / mm / yyyy)
- Sex ☐ Male ☐ Female
- Current Marital Status

☐ Married/Common-Law ☐ Separated/Divorced ☐ Single/Never Married
☐ Widowed ☐ Not answered

- Current Highest Level of Education

☐ No high school ☐ Some high school ☐ High school diploma
☐ Some post secondary ☐ Completed Post Secondary ☐ Not answered

- Current Employment Status

☐ Employed full-time ☐ Employed part-time ☐ Other, specify
☐ Unemployed ☐ Employed casual __________________________
☐ Retired ☐ On Disability ☐ Not answered

- Current Household Income *(before taxes & deductions)*

Although many health expenses are covered by health insurance, there is still a relationship between health and income. Please be assured that, like all other information you have provided, these answers will be kept strictly confidential.

- What is your best estimate of the total income, before taxes and deductions, of all household members from all sources in the past 12 months?

☐ < 15,000 ☐ 15,000 – 29,999 ☐ 30,000 – 49,999
☐ 50,000 – 79,999 ☐ ≥ 80,000 ☐ Not answered

Continue →

©2013 EPICORE Centre

EVOLUTION

Evaluating Self-Management and Educational Support in Extremely Obese Patients Awaiting Multidisciplinary Bariatric Care

Study ID ☐ - ☐☐☐
Site # Randomization #

Participant Initials ☐☐☐
First Middle Last

Demographics - continued

● Race *(check all that apply)*

☐ Canadian First Nations, Inuit or Métis

(A person having origins in any of the original peoples of Canada who maintains cultural identification through tribal affiliation or community recognition)

☐ American Indian or Alaskan Native

(A person having origins in any of the original peoples of North America who maintains cultural identification through tribal affiliation or community recognition)

☐ Asian

(A person having origins in any of the original peoples of the Far East, Southeast Asia or the Indian subcontinent including, eg. China, India, Japan, Korea, Malaysia, Pakistan, Philippine Islands, Cambodia, Thailand or Vietnam)

☐ Black or African American

(A person having origins in any of the black racial groups of Africa)

☐ White

(A person having origins in any of the original peoples of Europe, North Africa or the Middle East)

☐ Native Hawaiian or Other Pacific Islander

(A person having origins in any of the original peoples of Hawaii, Guam, Samoa or other Pacific Islands)

Continue →

©2013 EPICORE Centre

Fax completed forms to EPICORE Centre (780) 492-6059 or 1-888-215-5474

EVOLUTION

Evaluating Self-Management and Educational Support in Extremely Obese Patients Awaiting Multidisciplinary Bariatric Care

Study ID [ ] - [ ][ ][ ] (Site #, Randomization #) **Participant Initials** [ ][ ][ ] (First Middle Last)

Medical History/Obesity-Related Comorbidities *(check all that apply)*

- Do you currently have, any of the following medical conditions?

Impaired Glucose Tolerance *(Pre-Diabetes)*	☐ Yes ☐ No
Diabetes Mellitus	☐ Yes ☐ No
Hypertension *(High Blood Pressure)*	☐ Yes ☐ No
Dyslipidemia *(High Cholesterol)*	☐ Yes ☐ No
Cardiovascular Disease	☐ Yes ☐ No
**If Yes,** Coronary *(Heart Attack, Angina)*	☐ Yes ☐ No
Peripheral *(Decreased blood flow in leg arteries)*	☐ Yes ☐ No
Cerebral *(Stroke or TIA)*	☐ Yes ☐ No
Heart Failure	☐ Yes ☐ No
Sleep Apnea	☐ Yes ☐ No
**If Yes,** On CPAP	☐ Yes ☐ No
Asthma	☐ Yes ☐ No
Gastroesophageal Reflux Disease *(Heartburn)*	☐ Yes ☐ No
NAFLD *(Fatty Liver)*	☐ Yes ☐ No
Gallbladder Disease or Gallstones	☐ Yes ☐ No
Osteoarthritis *(Not Rheumatoid Arthritis)*	☐ Yes ☐ No
Chronic Renal Disorder *(Kidney Disease)*	☐ Yes ☐ No
Polycystic Ovary Syndrome	☐ Yes ☐ No
Hypothyroidism	☐ Yes ☐ No

Continue →

©2013 EPICORE Centre

EVOLUTION

Evaluating Self-Management and Educational Support in Extremely Obese Patients Awaiting Multidisciplinary Bariatric Care

Study ID ☐ - ☐☐☐ (Site # - Randomization #) **Participant Initials** ☐☐☐ (First Middle Last)

Medical History/Obesity-Related Comorbidities - continued

Cancer History	☐ Yes ☐ No
**If Yes,** Gastrointestinal *(esophagus, stomach, colon, rectal, pancreatic)*	☐ Yes ☐ No
Reproductive *(uterine, ovarian, prostate, breast)*	☐ Yes ☐ No
Other, specify ______________________________	☐ Yes ☐ No
Lymphedema	☐ Yes ☐ No
Mental Illness	☐ Yes ☐ No
**If Yes,** Depression	☐ Yes ☐ No
Bipolar Disorder	☐ Yes ☐ No
Abuse	☐ Yes ☐ No
**If Yes,** Sexual	☐ Yes ☐ No
Mental	☐ Yes ☐ No
Physical	☐ Yes ☐ No
Post Traumatic Stress Disorder	☐ Yes ☐ No
Binge Eating Disorder	☐ Yes ☐ No
Attention Deficit Disorder	☐ Yes ☐ No
Addiction	☐ Yes ☐ No
**If Yes,** Drug	☐ Yes ☐ No
Alcohol	☐ Yes ☐ No
Smoking	☐ Yes ☐ No
**If Yes,** ☐ Current	
☐ Former [Quit Date: _____ / __________] (mm / yyyy)	
Other	☐ Yes ☐ No
**If Yes,** specify ______________________________	

©2013 EPICORE Centre

Fax completed forms to EPICORE Centre (780) 492-6059 or 1-888-215-5474

EVOLUTION

Evaluating Self-Management and Educational Support in Extremely Obese Patients Awaiting Multidisciplinary Bariatric Care

Study ID [ ] - [ ][ ][ ] **Participant Initials** [ ][ ][ ]

Site # Randomization # First Middle Last

Blood Pressure/Heart Rate

- Average BP and Heart Rate *(recorded with Watch BP Monitor)*

Left Arm BP (*systolic/diastolic)*	Right Arm BP (*systolic/diastolic)*	Heart Rate
___ ___ ___ / ___ ___ ___ mmHg	___ ___ ___ / ___ ___ ___ mmHg	________ bpm

- Record all 3 readings *(recorded with Watch BP Monitor)*

#	Left Arm BP (*systolic/diastolic)*	Right Arm BP (*systolic/diastolic)*	Heart Rate
1	___ ___ ___ / ___ ___ ___ mmHg	___ ___ ___ / ___ ___ ___ mmHg	________ bpm
2	___ ___ ___ / ___ ___ ___ mmHg	___ ___ ___ / ___ ___ ___ mmHg	________ bpm
3	___ ___ ___ / ___ ___ ___ mmHg	___ ___ ___ / ___ ___ ___ mmHg	________ bpm

Form completed by ______________________ Signature ______________________ Date ____/____/_______
(please print name) dd mm yyyy

©2013 EPICORE Centre

EVOLUTION

Evaluating Self-Management and Educational Support in Extremely Obese Patients Awaiting Multidisciplinary Bariatric Care

Study ID [ ] - [ ][ ][ ]
Site # Randomization #

Participant Initials [ ][ ][ ]
First Middle Last

Blood Pressure/Heart Rate

Forearm BP

- **only record if unable to obtain upper arm BP**

Average BP and Heart Rate *(recorded with Watch BP Monitor)*

Left Arm BP (*systolic/diastolic)*	Right Arm BP (*systolic/diastolic)*	Heart Rate
___ ___ ___ / ___ ___ ___ mmHg	___ ___ ___ / ___ ___ ___ mmHg	________ bpm

- Record all 3 readings *(recorded with Watch BP Monitor)*

#	Left Arm BP (*systolic/diastolic)*	Right Arm BP (*systolic/diastolic)*	Heart Rate
1	___ ___ ___ / ___ ___ ___ mmHg	___ ___ ___ / ___ ___ ___ mmHg	________ bpm
2	___ ___ ___ / ___ ___ ___ mmHg	___ ___ ___ / ___ ___ ___ mmHg	________ bpm
3	___ ___ ___ / ___ ___ ___ mmHg	___ ___ ___ / ___ ___ ___ mmHg	________ bpm

Form completed by ______________________ Signature ______________________ Date ____/____/_______
(please print name) dd mm yyyy

©2013 EPICORE Centre

Fax completed forms to EPICORE Centre (780) 492-6059 or 1-888-215-5474

EVOLUTION

Evaluating Self-Management and Educational Support in Extremely Obese Patients Awaiting Multidisciplinary Bariatric Care

Study ID ☐ - ☐☐☐ **Participant Initials** ☐☐☐

Site # Randomization # First Middle Last

Timepoint ☐ **Baseline** ☐ **9 Month**

● Is participant **regularly** taking any **prescription** medications ☐ Yes ☐ No

If Yes, Total number of prescription medications ________

List prescription medications below *(generic name only)*

Generic Medication Name	For Coordinator Use ONLY Not for data entry **Dose**	**Frequency**	Total Daily Dose (mg)	Unable to enter into Database
________	________	________	________	☐
________	________	________	________	☐
________	________	________	________	☐
________	________	________	________	☐
________	________	________	________	☐
________	________	________	________	☐
________	________	________	________	☐
________	________	________	________	☐
________	________	________	________	☐
________	________	________	________	☐
________	________	________	________	☐
________	________	________	________	☐
________	________	________	________	☐

Continue →

©2013 EPICORE Centre

Fax completed forms to EPICORE Centre (780) 492-6059 or 1-888-215-5474

EVOLUTION

Evaluating Self-Management and Educational Support in Extremely Obese Patients Awaiting Multidisciplinary Bariatric Care

Study ID ☐ - ☐☐☐ **Participant Initials** ☐☐☐

Site # Randomization # First Middle Last

Timepoint ☐ **Baseline** ☐ **9 Month**

Current Medications - continued

● Medication Adherence

Which of the following categories best describes your use of your medications?

☐ Take all your pills
☐ Take 75-99% of your pills
☐ Take 50-74% of your pills
☐ Take less than 50% of your pills
☐ Take none of your pills
☐ Don't know

● Is participant taking any over-the-counter weight loss products ☐ Yes ☐ No

If Yes, How many ________

Brand Name(s)

1. ______________________________

2. ______________________________

3. ______________________________

Form completed by ______________________ Signature ______________________ Date ____/____/_______
(please print name) dd mm yyyy

©2013 EPICORE Centre

EVOLUTION

Evaluating Self-Management and Educational Support in Extremely Obese Patients Awaiting Multidisciplinary Bariatric Care

Study ID [ ] - [ ][ ][ ]
Site # Randomization #

Participant Initials [ ][ ][ ]
First Middle Last

Timepoint [ ] **Baseline** [ ] **9 Month**

At Baseline: labs must be within 3 months previous or 2 weeks after

At 9 Months: labs must be within 2 weeks before/after

Date of Collection ___ /___ /________
dd mm yyyy

Lab N/A	Lab Test	Lab Value	Unit of Collection	Same Date of Collection	If Date of Collection is different from above, enter below *(dd/mm/yyyy)*
☐	HbA1c	___ ___ • ___	%	☐	_____ / _____ / __________
☐	Creatinine	___ ___ ___	µmol/L	☐	_____ / _____ / __________
☐	GFR *(Calculated)*	___ ___	mL/min/1.73/m^2	☐	_____ / _____ / __________
☐	ALT	___ ___ ___	U/L	☐	_____ / _____ / __________

Fasting Lipid Panel:

☐	Total Cholesterol	___ • ___ ___	mmol/L	☐	_____ / _____ / __________
☐	Triglycerides	___ ___ • ___ ___	mmol/L	☐	_____ / _____ / __________
☐	LDL	___ • ___ ___	mmol/L	☐	_____ / _____ / __________
☐	HDL	___ • ___ ___	mmol/L	☐	_____ / _____ / __________
☐	Total: HDL Ratio	___ • ___		☐	_____ / _____ / __________

Form completed by ______________________ Signature ______________________ Date ____/____/_______
(please print name) dd mm yyyy

©2013 EPICORE Centre

Fax completed forms to EPICORE Centre (780) 492-6059 or 1-888-215-5474

EVOLUTION

Evaluating Self-Management and Educational Support in Extremely Obese Patients Awaiting Multidisciplinary Bariatric Care

Questionnaire Package

Dear Study Participant

These questionnaires ask about your health. Please select the answer that best describes how you feel. There are no right or wrong answers. No matter what answers you record, you are guaranteed the same treatment. You may leave an answer blank if you object to the question. If you have any questions or concerns, you may direct them to the research staff.

The information you provide will be kept confidential. Only the investigators will have access to the information collected in this study. Any report or presentation of this study will not identify you by name.

Thank you for your time

Date these questionnaires were completed

__________ / __________ / __________

Day Month Year

©2013 EPICORE Centre

EVOLUTION

Evaluating Self-Management and Educational Support in
Extremely Obese Patients Awaiting Multidisciplinary Bariatric Care

Study ID ☐ - ☐☐☐ **Participant Initials** ☐☐☐

Site # Randomization # First Middle Last

Visit ☐ **Baseline** ☐ **3 Month** ☐ **6 Month** ☐ **9 Month**

We would like to find out how satisfied you have felt with the health care you have been receiving.

How strongly do you AGREE or DISAGREE with this statement? On the line beside the statement, mark the checkbox below the opinion which is closest to your own view.

(Check one box ONLY)

		Strongly Agree	Agree	Uncertain	Disagree	Strongly Disagree
1.	The medical care I have been receiving is just about perfect	☐	☐	☐	☐	☐

©2013 EPICORE Centre

EVOLUTION

Evaluating Self-Management and Educational Support in Extremely Obese Patients Awaiting Multidisciplinary Bariatric Care

Study ID ☐ - ☐☐☐ (Site # / Randomization #) **Participant Initials** ☐☐☐ (First / Middle / Last)

Visit ☐ **Baseline** ☐ **3 Month** ☐ **6 Month** ☐ **9 Month**

This survey asks for your views about your health. This information will help keep track of how you feel and how well you are able to do your usual activities. For each one of the following questions, please mark the one checkbox that best describes your answer.

1. In general would you say your health is:

☐ Excellent ☐ Very Good ☐ Good ☐ Fair ☐ Poor

2. The following questions are about activities you might do during a typical day. Does your health now limit you in these activities? If so, how much?

a. <u>Moderate activities</u>, such as moving a table, pushing a vacuum cleaner, bowling or playing golf

☐ Yes, limited a lot ☐ Yes, limited a little ☐ No, not limited at all

b. Climbing <u>several</u> flights of stairs

☐ Yes, limited a lot ☐ Yes, limited a little ☐ No, not limited at all

3. During the past 4 weeks, how much of the time have you had any of the following problems with your work or other regular daily activities as a result of your physical health?

a. <u>Accomplished</u> less than you would like

☐ All the time ☐ Most of the time ☐ Some of the time ☐ A little of the time ☐ None of the time

b. Were limited in the <u>kind</u> of work or other activities

☐ All the time ☐ Most of the time ☐ Some of the time ☐ A little of the time ☐ None of the time

4. During the past 4 weeks, how much of the time have you had any of the following problems with your work or other regular daily activities as a result of any emotional problems (such as feeling depressed or anxious)?

a. <u>Accomplished</u> less than you would like

☐ All the time ☐ Most of the time ☐ Some of the time ☐ A little of the time ☐ None of the time

b. Did work or other activities less <u>carefully than usual</u>

☐ All the time ☐ Most of the time ☐ Some of the time ☐ A little of the time ☐ None of the time

©2013 EPICORE Centre

EVOLUTION

Evaluating Self-Management and Educational Support in Extremely Obese Patients Awaiting Multidisciplinary Bariatric Care

Study ID ☐ - ☐☐☐ (Site #, Randomization #) **Participant Initials** ☐☐☐ (First, Middle, Last)

Visit ☐ **Baseline** ☐ **3 Month** ☐ **6 Month** ☐ **9 Month**

---

5. During the past 4 weeks, how much did pain interfere with your normal work (including both work outside the home and housework)?

☐ Not at all ☐ A little bit ☐ Moderately ☐ Quite a bit ☐ Extremely

6. These questions are about how you feel and how things have been with you during the past 4 weeks. For each question, please give the one answer that comes closest to the way you have been feeling. How much of the time during the past 4 weeks…

a. Have you felt calm and peaceful?

☐ All the time ☐ Most of the time ☐ Some of the time ☐ A little of the time ☐ None of the time

b. Did you have a lot of energy?

☐ All the time ☐ Most of the time ☐ Some of the time ☐ A little of the time ☐ None of the time

c. Have felt downhearted and depressed?

☐ All the time ☐ Most of the time ☐ Some of the time ☐ A little of the time ☐ None of the time

7. During the past 4 weeks, how much of the time has your physical health or emotional problems interfered with your social activities (like visiting with friends, relatives, etc)?

☐ All the time ☐ Most of the time ☐ Some of the time ☐ A little of the time ☐ None of the time

©2013 EPICORE Centre

EVOLUTION

Evaluating Self-Management and Educational Support in Extremely Obese Patients Awaiting Multidisciplinary Bariatric Care

Study ID ☐ - ☐☐☐ **Participant Initials** ☐☐☐

Site # Randomization # First Middle Last

Visit ☐ **Baseline** ☐ **3 Month** ☐ **6 Month** ☐ **9 Month**

Health Questionnaire

English version for Canada

Canada (English) © 1999 EuroQol Group. EQ-5D™ is a trade mark of the EuroQol Group

©2013 EPICORE Centre

EVOLUTION

Evaluating Self-Management and Educational Support in Extremely Obese Patients Awaiting Multidisciplinary Bariatric Care

Study ID ☐ - ☐☐☐ **Participant Initials** ☐☐☐

Site # Randomization # First Middle Last

Visit ☐ **Baseline** ☐ **3 Month** ☐ **6 Month** ☐ **9 Month**

By placing a check-mark in one box in each group below, please indicate which statements best describe your own state of health today.

Mobility

I have no problems in walking about ☐

I have some problems in walking about ☐

I am confined to bed ☐

Self-Care

I have no problems with self-care ☐

I have some problems washing or dressing myself ☐

I am unable to wash or dress myself ☐

Usual Activities (*e.g. work, study, housework, family or leisure activities)*

I have no problems with performing my usual activities ☐

I have some problems with performing my usual activities ☐

I am unable to perform my usual activities ☐

Pain/Discomfort

I have no pain or discomfort ☐

I have moderate pain or discomfort ☐

I have extreme pain or discomfort ☐

Anxiety/Depression

I am not anxious or depressed ☐

I am moderately anxious or depressed ☐

I am extremely anxious or depressed ☐

Canada (English) © 1999 EuroQol Group. EQ-5D™ is a trade mark of the EuroQol Group

©2013 EPICORE Centre

EVOLUTION

Evaluating Self-Management and Educational Support in
Extremely Obese Patients Awaiting Multidisciplinary Bariatric Care

Study ID ☐ - ☐☐☐ **Participant Initials** ☐☐☐

Site # Randomization # First Middle Last

Visit ☐ **Baseline** ☐ **3 Month** ☐ **6 Month** ☐ **9 Month**

To help people say how good or bad their state of health is, we have drawn a scale (rather like a thermometer) on which the best state you can imagine is marked 100 and the worst state you can imagine is marked 0.

We would like you to indicate on this scale how good or bad your own health is today, in your opinion. Please do this by drawing a line from the box below to whichever point on the scale indicates how good or bad your state of health is today.

Your own state of health today

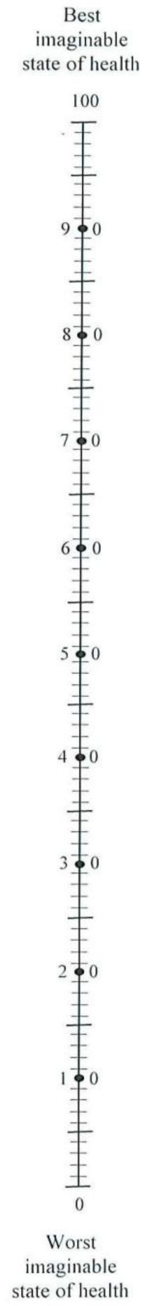

Canada (English) © 1999 EuroQol Group. EQ-5D™ is a trade mark of the EuroQol Group

©2013 EPICORE Centre

Fax completed forms to EPICORE Centre (780) 492-6059 or 1-888-215-5474

EVOLUTION

Evaluating Self-Management and Educational Support in Extremely Obese Patients Awaiting Multidisciplinary Bariatric Care

Study ID ☐ - ☐☐☐ **Participant Initials** ☐☐☐

Site # Randomization # First Middle Last

Visit ☐ **Baseline** ☐ **3 Month** ☐ **6 Month** ☐ **9 Month**

Over the **last 2 weeks**, how often have you been bothered by any of the following problems? *Mark one box only in answer to each question*	Not at all	Several days	More than half the days	Nearly every day
**1. Little interest or pleasure in doing things**	☐	☐	☐	☐
**2. Feeling down, depressed, or hopeless**	☐	☐	☐	☐
**3. Trouble falling or staying asleep or sleeping too much**	☐	☐	☐	☐
**4. Feeling tired or having little energy**	☐	☐	☐	☐
**5. Poor appetite or overeating**	☐	☐	☐	☐
**6. Feeling bad about yourself – or that you are a failure or have let yourself or your family down**	☐	☐	☐	☐
**7. Trouble concentrating on things, such as reading the newspaper or watching television**	☐	☐	☐	☐
**8. Moving or speaking so slowly that other people could have noticed? Or the opposite – being so fidgety or restless that you have been moving around a lot more than usual**	☐	☐	☐	☐

If you checked off any problems, how difficult have these problems made it for you to do your work, take care of things at home, or get along with other people?

Not difficult at all	Somewhat difficult	Very difficult	Extremely difficult
☐	☐	☐	☐

Developed by Drs. Robert L. Spitzer, Janet B.W. Williams, Hurt Kroenke and colleagues, with an educational grant from Pfizer Inc.
No permission required to reproduce, translate, display or distribute

©2013 EPICORE Centre

Fax completed forms to EPICORE Centre (780) 492-6059 or 1-888-215-5474

EVOLUTION

Evaluating Self-Management and Educational Support in
Extremely Obese Patients Awaiting Multidisciplinary Bariatric Care

Study ID ☐ - ☐☐☐ **Participant Initials** ☐☐☐

Site # Randomization # First Middle Last

Visit ☐ **Baseline** ☐ **3 Month** ☐ **6 Month** ☐ **9 Month**

- **How ready are you to make changes in your life to reduce your weight?**

Please mark an 'x' on the line below to indicate your response

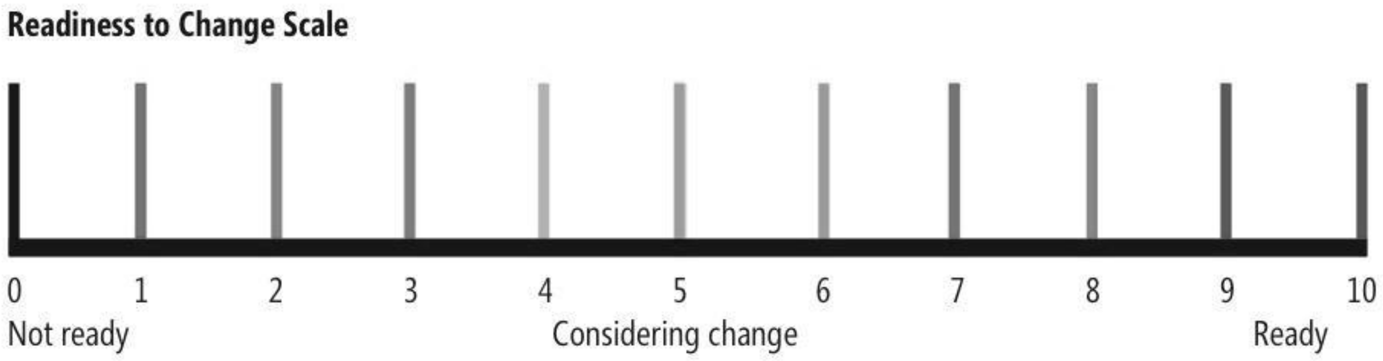

©2013 EPICORE Centre

Fax completed forms to EPICORE Centre (780) 492-6059 or 1-888-215-5474

EVOLUTION

Evaluating Self-Management and Educational Support in Extremely Obese Patients Awaiting Multidisciplinary Bariatric Care

Study ID [ ] **-** [ ][ ][ ] **Participant Initials** [ ][ ][ ]

Site # Randomization # First Middle Last

Visit [ ] **Baseline** [ ] **3 Month** [ ] **6 Month** [ ] **9 Month**

Listed below are a number of situations that lead some people to eat. We would like to know **how confident** you are that you would **not** eat in each situation.

Circle the number that best describes your feelings of **confidence to not eat food in each situation**

1. I can resist eating when I am anxious (nervous).

**0**	**1**	**2**	**3**	**4**	**5**	**6**	**7**	**8**	**9**
Not confident			Somewhat Confident					Very Confident	

2. I can control my eating on the weekends.

**0**	**1**	**2**	**3**	**4**	**5**	**6**	**7**	**8**	**9**
Not confident			Somewhat Confident					Very Confident	

3. I can resist eating even when I have to say “NO” to others.

**0**	**1**	**2**	**3**	**4**	**5**	**6**	**7**	**8**	**9**
Not confident			Somewhat Confident					Very Confident	

4. I can resist eating when I feel physically run down.

**0**	**1**	**2**	**3**	**4**	**5**	**6**	**7**	**8**	**9**
Not confident			Somewhat Confident					Very Confident	

5. I can resist eating when I am watching TV.

**0**	**1**	**2**	**3**	**4**	**5**	**6**	**7**	**8**	**9**
Not confident			Somewhat Confident					Very Confident	

Continue →

©2013 EPICORE Centre
Fax completed forms to EPICORE Centre (780) 492-6059 or 1-888-215-5474

EVOLUTION

Evaluating Self-Management and Educational Support in Extremely Obese Patients Awaiting Multidisciplinary Bariatric Care

Study ID ☐ - ☐☐☐ **Participant Initials** ☐☐☐

Site # Randomization # First Middle Last

Visit ☐ **Baseline** ☐ **3 Month** ☐ **6 Month** ☐ **9 Month**

Circle the number that best describes your feelings of **confidence to not eat food in each situation**

6. I can resist eating when I am depressed (or down).

0 1 2 3 4 5 6 7 8 9

Not confident Somewhat Confident Very Confident

7. I can resist eating when there are many different kinds of food available.

0 1 2 3 4 5 6 7 8 9

Not confident Somewhat Confident Very Confident

8. I can resist eating even when I feel it's impolite to refuse a second helping.

0 1 2 3 4 5 6 7 8 9

Not confident Somewhat Confident Very Confident

9. I can resist eating even when I have a headache.

0 1 2 3 4 5 6 7 8 9

Not confident Somewhat Confident Very Confident

10. I can resist eating when I am reading.

0 1 2 3 4 5 6 7 8 9

Not confident Somewhat Confident Very Confident

11. I can resist eating when I am angry (or irritable).

0 1 2 3 4 5 6 7 8 9

Not confident Somewhat Confident Very Confident

Continue →

©2013 EPICORE Centre

EVOLUTION

Evaluating Self-Management and Educational Support in
Extremely Obese Patients Awaiting Multidisciplinary Bariatric Care

Study ID ☐ - ☐☐☐ **Participant Initials** ☐☐☐

Site # Randomization # First Middle Last

Visit ☐ **Baseline** ☐ **3 Month** ☐ **6 Month** ☐ **9 Month**

Circle the number that best describes your feelings of **confidence to not eat food in each situation**

12. I can resist eating even when I am at a party.

0	1	2	3	4	5	6	7	8	9
Not confident			Somewhat Confident					Very Confident	

13. I can resist eating even when others are pressuring me to eat.

0	1	2	3	4	5	6	7	8	9
Not confident			Somewhat Confident					Very Confident	

14. I can resist eating when I am in pain.

0	1	2	3	4	5	6	7	8	9
Not confident			Somewhat Confident					Very Confident	

15. I can resist eating just before going to bed.

0	1	2	3	4	5	6	7	8	9
Not confident			Somewhat Confident					Very Confident	

16. I can resist eating when I have experienced failure.

0	1	2	3	4	5	6	7	8	9
Not confident			Somewhat Confident					Very Confident	

17. I can resist eating even when high-calorie foods are available.

0	1	2	3	4	5	6	7	8	9
Not confident			Somewhat Confident					Very Confident	

Continue →

©2013 EPICORE Centre

EVOLUTION

Evaluating Self-Management and Educational Support in Extremely Obese Patients Awaiting Multidisciplinary Bariatric Care

Study ID ☐ - ☐☐☐ **Participant Initials** ☐☐☐

Site # Randomization # First Middle Last

Visit ☐ **Baseline** ☐ **3 Month** ☐ **6 Month** ☐ **9 Month**

Circle the number that best describes your feelings of **confidence to not eat food in each situation**

18. I can resist eating even when I think others will be upset if I don't eat.

0	1	2	3	4	5	6	7	8	9
Not confident			Somewhat Confident					Very Confident	

19. I can resist eating when I feel uncomfortable.

0	1	2	3	4	5	6	7	8	9
Not confident			Somewhat Confident					Very Confident	

20. I can resist eating when I am happy.

0	1	2	3	4	5	6	7	8	9
Not confident			Somewhat Confident					Very Confident	

Clark, M.M.; Abrams, D.B.; Niaura, R.S.; Eaton, C.A.; and Rossi, J.S. (1991). Self-efficacy in weight management. Journal of Consulting and Clinical Psychology, 59(5), 739-744

©2013 EPICORE Centre

Fax completed forms to EPICORE Centre (780) 492-6059 or 1-888-215-5474

EVOLUTION

Evaluating Self-Management and Educational Support in
Extremely Obese Patients Awaiting Multidisciplinary Bariatric Care

Study ID ☐ - ☐☐☐ **Participant Initials** ☐☐☐

Site # Randomization # First Middle Last

Visit ☐ **Baseline** ☐ **3 Month** ☐ **6 Month** ☐ **9 Month**

1. How often do you have problems learning about your medical condition because of difficulty understanding written information?

- ☐ All of the time
- ☐ Most of the time
- ☐ Some of the time
- ☐ A little of the time
- ☐ None of the time

2. How confident are you filling out medical forms by yourself?

- ☐ All of the time
- ☐ Most of the time
- ☐ Some of the time
- ☐ A little of the time
- ☐ None of the time

3. How often do you have someone like a family member, friend, hospital or clinic worker or caregiver, help you read health plan materials (such as written information about your health or care you are offered)?

- ☐ All of the time
- ☐ Most of the time
- ☐ Some of the time
- ☐ A little of the time
- ☐ None of the time

Chew LD, et al. J Gen Intern Med 23(5):561–6

©2013 EPICORE Centre

EVOLUTION

Evaluating Self-Management and Educational Support in Extremely Obese Patients Awaiting Multidisciplinary Bariatric Care

Study ID ☐ - ☐☐☐ **Participant Initials** ☐☐☐

Site # Randomization # First Middle Last

Visit ☐ **Baseline** ☐ **3 Month** ☐ **6 Month** ☐ **9 Month**

We would like to find out how satisfied you have felt with the health care you have been receiving.

How strongly do you AGREE or DISAGREE with this statement? On the line beside the statement, mark the checkbox below the opinion which is closest to your own view.

(Check one box ONLY)

		Strongly Agree	Agree	Uncertain	Disagree	Strongly Disagree
1.	I am dissatisfied with some things about the medical care I received	☐	☐	☐	☐	☐

©2013 EPICORE Centre

EVOLUTION

Evaluating Self-Management and Educational Support in Extremely Obese Patients Awaiting Multidisciplinary Bariatric Care

FACSIMILE TRANSMITTAL SHEET

To: Dr. ______________________________ **Fax #** ______________________________

Re: ______________________________ **Date** ______________________________

Dear Colleague,

The above-named patient is wait-listed for Bariatric Care and has been enrolled in the CIHR-funded *EVOLUTION Trial*. This 9-month randomized controlled trial is being performed in partnership with Alberta Health Services and will compare three different educational interventions designed to improve patients' success once in bariatric care. Patients will receive three months of education and then be followed within the bariatric clinic for six months. Outcomes include body weight, quality of life and costs.

Patients will have bloodwork at baseline and 9 months *(fasting lipids, creatinine, A1c).* Please note that to avoid unnecessary duplication of testing, this bloodwork will be available on *NetCare.*

Best Regards,

Raj Padwal, MD, FRCP(C)
Principal Investigator
EVOLUTION Trial

©2013 EPICORE Centre

EVOLUTION

Evaluating Self-Management and Educational Support in Extremely Obese Patients Awaiting Multidisciplinary Bariatric Care

Study ID ☐ - ☐☐☐ (Site #, Randomization #) **Participant Initials** ☐☐☐ (First, Middle, Last)

Visit ☐ **3 Month** ☐ **6 Month** ☐ **9 Month**

- Were you able to conduct this **Follow Up** visit ☐ Yes ☐ No

If Yes, Date of visit _____ / _____ / _________ (dd mm yyyy)

If No, Specify reason

☐ Missed Visit
☐ Early Withdrawal → complete ***Early Withdrawal*** form
☐ Death → complete ***Early Withdrawal*** form
☐ Other

Weight Wise Clinic

- **Since the last contact,** has participant started **treatment** *(not assessment)* ☐ Yes ☐ No

If Yes, Date of first **treatment** *(not assessment)* visit _____ / _____ / _________ (dd mm yyyy)

- Clinic Interventions
- Has participant utilized any of the following services at the *Weight Wise Clinic*

Dietitian	☐ Yes ☐ No	If Yes, # of Visits ________
Physiotherapy	☐ Yes ☐ No	If Yes, # of Visits ________
Psychologist *(alone or group therapy)*	☐ Yes ☐ No	If Yes, # of Visits ________
Occupational Therapy	☐ Yes ☐ No	If Yes, # of Visits ________
Psychiatrist	☐ Yes ☐ No	If Yes, # of Visits ________
Social Worker	☐ Yes ☐ No	If Yes, # of Visits ________
Physician	☐ Yes ☐ No	If Yes, # of Visits ________
Sleep Specialist	☐ Yes ☐ No	If Yes, # of Visits ________

Continue →

©2013 EPICORE Centre

EVOLUTION

Evaluating Self-Management and Educational Support in Extremely Obese Patients Awaiting Multidisciplinary Bariatric Care

Study ID ☐ - ☐☐☐ (Site #, Randomization #) **Participant Initials** ☐☐☐ (First, Middle, Last)

Visit ☐ **3 Month** ☐ **6 Month** ☐ **9 Month**

Weight Wise Group Workshops

To be completed for all participants (irrespective of group assignment)

● **Since the last contact,** Has participant completed any *In-person Weight Wise Modules* ☐ Yes ☐ No

If Yes, Record workshops attended

Getting Started: Planning for Success	☐ Yes ☐ No
Lifestyle Change: A Toolkit for Success	☐ Yes ☐ No
Nutrition: Finding Balance: The Role of Calories in Weight Management	☐ Yes ☐ No
Managing Hunger and Appetite	☐ Yes ☐ No
Moving Matters: Including Physical Activity in your Day	☐ Yes ☐ No
Nutrition: The Truth about What Works in Weight Management	☐ Yes ☐ No
Nutrition: I know I should Eat Healthy, But How?	☐ Yes ☐ No
Nutrition: Eating Away from Home and during Special Occasions	☐ Yes ☐ No
Minding Stress: Effectively Reduce and Manage the Stress in your Life	☐ Yes ☐ No

Continue →

©2013 EPICORE Centre

EVOLUTION

Evaluating Self-Management and Educational Support in Extremely Obese Patients Awaiting Multidisciplinary Bariatric Care

Study ID ☐ **-** ☐☐☐ **Participant Initials** ☐☐☐

Site # Randomization # First Middle Last

Visit ☐ **3 Month** ☐ **6 Month** ☐ **9 Month**

Weight Wise On-Line Modules

To be completed ONLY for participants in the Web-based Module Treatment Assignment

- **Since the last contact,** Has participant completed any *Web-based Weight Wise Modules* ☐ Yes ☐ No

If Yes, Record modules completed:

Getting Started: Planning for Success	☐ Yes ☐ No
Getting Started: Benefits & Challenges	☐ Yes ☐ No
Getting Started: Skills for Weight Management Success	☐ Yes ☐ No
Finding Balance: The Role of Calories in Weight Management: – Calories & Diets	☐ Yes ☐ No
Finding Balance: The Role of Calories in Weight Management: – Top 5 Calorie Culprits & Tips for Reducing Calories	☐ Yes ☐ No
Managing Hunger & Appetite: Managing Hunger	☐ Yes ☐ No
Managing Hunger & Appetite: Controlling Your Appetite	☐ Yes ☐ No
Moving Matters: How does moving matter?	☐ Yes ☐ No
Moving Matters: Help me get moving! I'm ready!	☐ Yes ☐ No
More on Nutrition: Transform your Eating for Weight Loss	☐ Yes ☐ No
More on Nutrition: Meal planning Tips and Label Reading	☐ Yes ☐ No
More on Nutrition: Eating Out and Special Occasions	☐ Yes ☐ No
A Good Night's Sleep	☐ Yes ☐ No

Continue →

©2013 EPICORE Centre

EVOLUTION

Evaluating Self-Management and Educational Support in Extremely Obese Patients Awaiting Multidisciplinary Bariatric Care

Study ID ☐ - ☐☐☐ **Participant Initials** ☐☐☐

Site # Randomization # First Middle Last

Visit ☐ **3 Month** ☐ **6 Month** ☐ **9 Month**

Demographics

● Current Marital Status

☐ Married/Common-Law ☐ Separated/Divorced ☐ Single/Never Married

☐ Widowed ☐ Not answered

Blood Pressure/Heart Rate

● Average BP and Heart Rate *(recorded with Watch BP Monitor)*

Left Arm BP (*systolic/diastolic)*	Right Arm BP (*systolic/diastolic)*	Heart Rate
___ ___ ___ / ___ ___ ___ mmHg	___ ___ ___ / ___ ___ ___ mmHg	_________ bpm

● Record all 3 readings *(recorded with Watch BP Monitor)*

#	Left Arm BP (*systolic/diastolic)*	Right Arm BP (*systolic/diastolic)*	Heart Rate
1	___ ___ ___ / ___ ___ ___ mmHg	___ ___ ___ / ___ ___ ___ mmHg	_________ bpm
2	___ ___ ___ / ___ ___ ___ mmHg	___ ___ ___ / ___ ___ ___ mmHg	_________ bpm
3	___ ___ ___ / ___ ___ ___ mmHg	___ ___ ___ / ___ ___ ___ mmHg	_________ bpm

Anthropometric Measures

**Weight**	_________ kg

Continue →

©2013 EPICORE Centre

EVOLUTION

Evaluating Self-Management and Educational Support in Extremely Obese Patients Awaiting Multidisciplinary Bariatric Care

Study ID ☐ - ☐☐☐ **Participant Initials** ☐☐☐

Site # Randomization # First Middle Last

Visit ☐ **3 Month** ☐ **6 Month** ☐ **9 Month**

Blood Pressure/Heart Rate

Forearm BP

- **only record if unable to obtain upper arm BP**

Average BP and Heart Rate *(recorded with Watch BP Monitor)*

Left Arm BP (*systolic/diastolic)*	Right Arm BP (*systolic/diastolic)*	Heart Rate
___ ___ ___ / ___ ___ ___ mmHg	___ ___ ___ / ___ ___ ___ mmHg	________ bpm

- Record all 3 readings *(recorded with Watch BP Monitor)*

#	Left Arm BP (*systolic/diastolic)*	Right Arm BP (*systolic/diastolic)*	Heart Rate
1	___ ___ ___ / ___ ___ ___ mmHg	___ ___ ___ / ___ ___ ___ mmHg	________ bpm
2	___ ___ ___ / ___ ___ ___ mmHg	___ ___ ___ / ___ ___ ___ mmHg	________ bpm
3	___ ___ ___ / ___ ___ ___ mmHg	___ ___ ___ / ___ ___ ___ mmHg	________ bpm

Form completed by ______________________ Signature ______________________ Date ____/____/________

(please print name) dd mm yyyy

©2013 EPICORE Centre

Fax completed forms to EPICORE Centre (780) 492-6059 or 1-888-215-5474

EVOLUTION

Evaluating Self-Management and Educational Support in Extremely Obese Patients Awaiting Multidisciplinary Bariatric Care

Study ID ☐ - ☐☐☐ **Participant Initials** ☐☐☐

Site # Randomization # First Middle Last

Visit ☐ **3 Month** ☐ **6 Month** ☐ **9 Month**

Smoking History

- **Since the last contact,** Has there been a change in smoking status ☐ Yes ☐ No

  If Yes, Type of Change: ☐ Restarted ☐ Quit

Surgery

- **Since the last contact,** Has participant had bariatric surgery ☐ Yes ☐ No

  If Yes, complete ***Bariatric Surgery*** form

Weight-Wise Clinic Discharge

- **Since the last contact,** Was participant discharged from the ***Weight Wise Clinic*** ☐ Yes ☐ No

  If Yes, Complete ***Weight Wise Clinic Discharge*** form

Form completed by ______________________ Signature ______________________ Date ____/____/_______
(please print name) dd mm yyyy

©2013 EPICORE Centre

Fax completed forms to EPICORE Centre (780) 492-6059 or 1-888-215-5474

EVOLUTION

Evaluating Self-Management and Educational Support in
Extremely Obese Patients Awaiting Multidisciplinary Bariatric Care

Study ID [ ] - [ ][ ][ ]
Site # Randomization #

Participant Initials [ ][ ][ ]
First Middle Last

This form is to be completed ONLY for patients discharged from the *Weight Wise* Clinic

● Date of *Weight Wise* Clinic Discharge _____ / _____ / _________
dd mm yyyy

● Reason for discharge *(check all that apply)*

- ☐ Self
- ☐ Treatment plan forwarded to community health care provider
- ☐ Non-compliant
- ☐ Referred elsewhere *(i.e. specialist)*
- ☐ Other, specify ____

Form completed by ______________________ Signature ______________________ Date ____/____/_______
(please print name) dd mm yyyy

©2013 EPICORE Centre

Fax completed forms to EPICORE Centre (780) 492-6059 or 1-888-215-5474

EVOLUTION

Evaluating Self-Management and Educational Support in Extremely Obese Patients Awaiting Multidisciplinary Bariatric Care

Study ID ☐ - ☐☐☐ **Participant Initials** ☐☐☐

Site # Randomization # First Middle Last

Visit ☐ **3 Month** ☐ **6 Month** ☐ **9 Month**

- Date of Early Withdrawal _______ /_______ /_________
  dd mm yyyy

- Reason for Early Withdrawal *(check only one)*

  ☐ No longer wishes to participate

  ☐ Deceased

  Date of death _______ /_______ /_________
  dd mm yyyy

  ☐ Lost to follow up

  Date of last contact _______ /_______ /_________
  dd mm yyyy

  Number of attempts made to contact participant ________

  Not for Data Entry

  Record of attempts to contact participant

  1. _______ /_______ /_________
     dd mm yyyy
  2. _______ /_______ /_________
     dd mm yyyy
  3. _______ /_______ /_________
     dd mm yyyy

  ☐ Other, specify reason ______________________________

Form completed by ____________________ Signature ____________________ Date ____/____/_______
(please print name) dd mm yyyy

©2013 EPICORE Centre

Fax completed forms to EPICORE Centre (780) 492-6059 or 1-888-215-5474
